# Skeletal remains of the oldest known pseudocoracid shark *Pseudocorax kindlimanni* sp. nov. (Chondrichthyes, Lamniformes) from the Late Cretaceous of Lebanon

**DOI:** 10.1016/j.cretres.2021.104842

**Published:** 2021-09

**Authors:** Patrick L. Jambura, Sebastian Stumpf, Jürgen Kriwet

**Affiliations:** University of Vienna, Department of Palaeontology, Althanstraβe 14, 1090, Vienna, Austria

**Keywords:** Chondrichthyes, Elasmobranchii, Pseudocoracidae, Heterodonty, Mesozoic, Haqel

## Abstract

A new fossil mackerel shark, *Pseudocorax kindlimanni* sp. nov. (Lamniformes, Pseudocoracidae), is described from the Cenomanian Konservat-Lagerstätte of Haqel, Lebanon. The new species is based on the most complete fossil of this group to date, which comprises an associated tooth set of 70 teeth, six articulated vertebral centra, numerous placoid scales and pieces of unidentifiable mineralized cartilage. The dentition of *P*. *kindlimanni* sp. nov. is marked by a high degree of monognathic heterodonty but does not exhibit the characteristic “lamnoid tooth pattern” known from other macrophagous lamniform sharks. In addition, *P*. *kindlimanni* sp. nov. shows differences in tooth microstructure and vertebral centrum morphology compared to other lamniform sharks. These variations, however, are also known from other members of this order and do not warrant the assignment of *Pseudocorax* outside the lamniform sharks. The new fossil is the oldest known pseudocoracid shark and pushes the origin of this group back into the Cenomanian, a time when lamniform sharks underwent a major diversification. This radiation resulted not only in high species diversity, but also in the development of a diverse array of morphological traits and adaptation to different ecological niches. *Pseudocorax kindlimanni* sp. nov. was a small, active predator capable of fast swimming, and it occupied the lower trophic levels of the marine food web in the Late Cretaceous.

## 1 Introduction

The Cretaceous represents a fundamental stage for the early evolution and adaptive radiation of numerous marine clades, including marine reptiles (Nicholson et al., 2015), bony fish (Guinot and Cavin, 2016), and elasmobranchs (sharks and rays; Underwood, 2006; Guinot and Cavin, 2016). Among sharks, the youngest extant orders emerged during this period, namely sawsharks, Pristio-phoriformes (Nevatte and Williamson, 2020) and dogfish sharks, Squaliformes (Kriwet and Klug, 2009). The timing of the origin of lamniform sharks is still debated, with mounting evidence that the Middle–Late Jurassic genus *Palaeocarcharias* de Beaumont, 1960 represents the oldest member of this group (Jambura et al., 2019). However, the impact the Cretaceous Period had on this group remains unquestioned. Various basal lamniforms occurred during the Valanginian–Barremian (Rees, 2005; Kriwet et al., 2008; CarrilloBriceño et al., 2019) that subsequently experienced a dramatic diversification during the Cenomanian (Underwood, 2006; Guinot, 2013; Guinot and Cavin, 2016), and culminated in lamniform sharks becoming a dominant group during the Cretaceous and Palaeogene. Lamniform diversity declined during the Neogene, and 15 taxa within seven different families occur today (Guinot and Cavin, 2016; Condamine et al., 2019).

Due to their cartilaginous skeleton, the preservation potential for these elasmobranch remains is generally low, resulting in a fossil record predominantly consisting of isolated teeth. Although shark teeth are considered to be diagnostic at species level, the presence of heterodonty (=different morphotypes within a species’ dentition) can hamper the ability to accurately identify and erect nominal species based on isolated teeth. Even though a number of new analytical methods have been developed to improve modelling biodiversity curves and avoid biases in the fossil record, the reconstruction of the evolutionary history of biological groups still faces major challenges. This is also true for lamniform sharks, and their species diversity through time remains difficult to assess due to insufficient descriptions and illustrations of different nominal species, and difficulties in assessing intraspecific variation (Siverson et al., 2007; Siversson et al., 2015).

Pseudocoracidae is a group of small lamniform sharks, whose members are primarily represented by isolated teeth from the Turonian to Maastrichtian of the northern hemisphere (Cappetta, 2012). Herein we describe the first skeletal remains of a new pseudocoracid shark from the Cenomanian of Lebanon, *Pseudocorax kindlimanni* sp. nov. This new specimen is the most complete fossil of this group to date and provides important insights into the dentition, ecology, stratigraphic range, and early evolution of pseudocoracid sharks. Furthermore, the fossil emphasizes the significance of the Cenomanian as a time of major radiation of lamniform sharks (and fishes in general) and development of a number of novel specializations and ecomorphotypes of which some are present to this day.

## 2 Geological setting

**Fig. 1:**
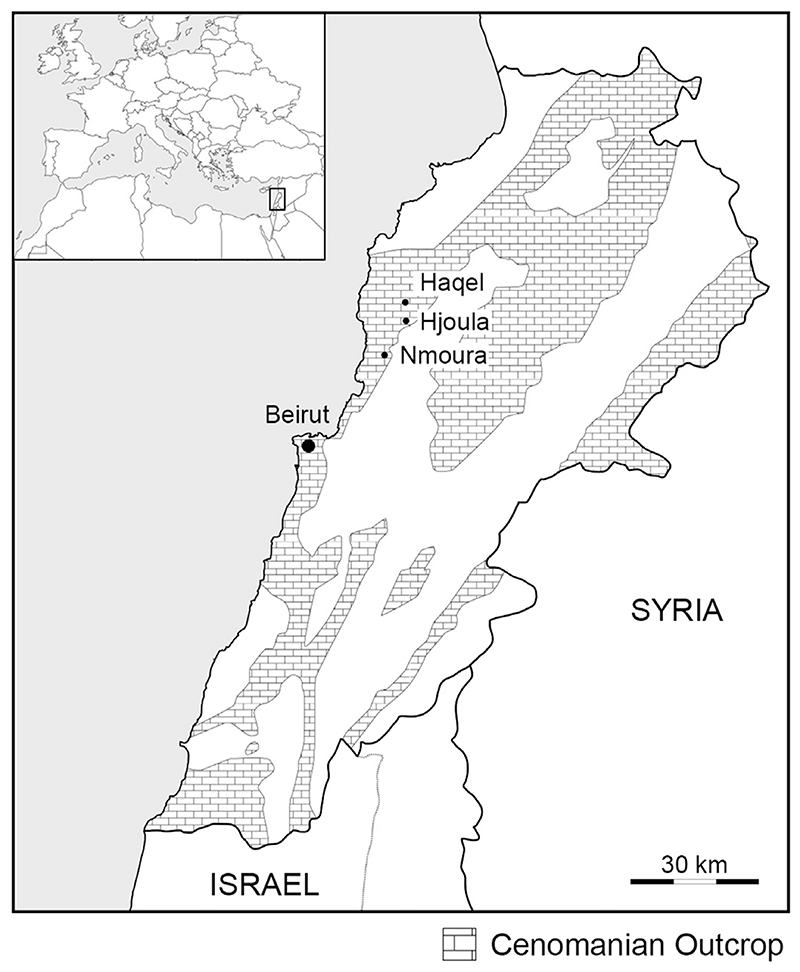
Locality map showing the position of the Lebanese Konservat-Lagerstätten of Haqel, Hjoula, and Nmoura. The distribution of Cenomanian outcrops in Lebanon is based on Forey et al. (2003).

The skeletal remains are embedded in a single limestone slab recovered from the Sannine Limestone (Upper Cretaceous) of Haqel, Lebanon. Haqel is one of the most famous Konservat-Lagerstätten in Lebanon, along with Hjoula, Nmoura, and Sahel Alma. It is located about 10 km inland from the seaport of Byblos and 45 km NE of Beirut (Fig. 1). The fish beds in Haqel were deposited in small basins only a few hundred meters across, probably representing sinkholes formed through tectonic activity on the contemporary seafloor at the outer margin of the continental shelf, along the south-western margin of the Tethys (Hückel, 1970; Hemleben, 1977). Although there is general agreement about the Cenomanian age of Haqel (between 100.5 ± 0.9 and 93.9 ± 0.8 Ma), precise dating varies according to different authors. Hückel (1970) conducted the most comprehensive sedimentological study of Haqel and divided the Sannine Limestone into seven lithological divisions. The fish-bearing layer of Haqel (layer 5) also contains the foraminiferan, *Orbitolina concava* (Lamarck, 1816), and the index ammonite, *Mantelliceras mantelli* (Sowerby, 1814), as reported by Zumoffen (1926) and therefore was assigned to the lower Cenomanian. This was in agreement with the findings of Saint-Marc (1974) and Dubertret (1963), although it is unclear on what evidence the latter author based his assumption (Forey et al., 2003). However, the occurrence of *Mantelliceras mantelli* is regarded doubtful, as this species has been used for various taxa within the family Acanthoceratidae de Grossouvre, 1894, and specimens have never been figured (Wippich and Lehmann, 2004). Other studies proposed either a middle Cenomanian age (Patterson, 1967) or late Cenomanian age (d’Erasmo, 1946; Arambourg, 1954; Hemleben, 1977). More recently, an early late Cenomanian age was supported by Wippich and Lehmann (2004) based on the presence of the heteromorphic ammonite, *Allocrioceras* cf. *annulatum* (Shumard, 1860), which is also known from the *Sciponoceras gracile* Zone of the Western Interior of the United States of America as well as the OAE2 interval of the Pecínov quarry in the Bohemian Cretaceous Basin of the Czech Republic (Košťák et al., 2018).

## 3 Materials and methods

**Fig. 2:**
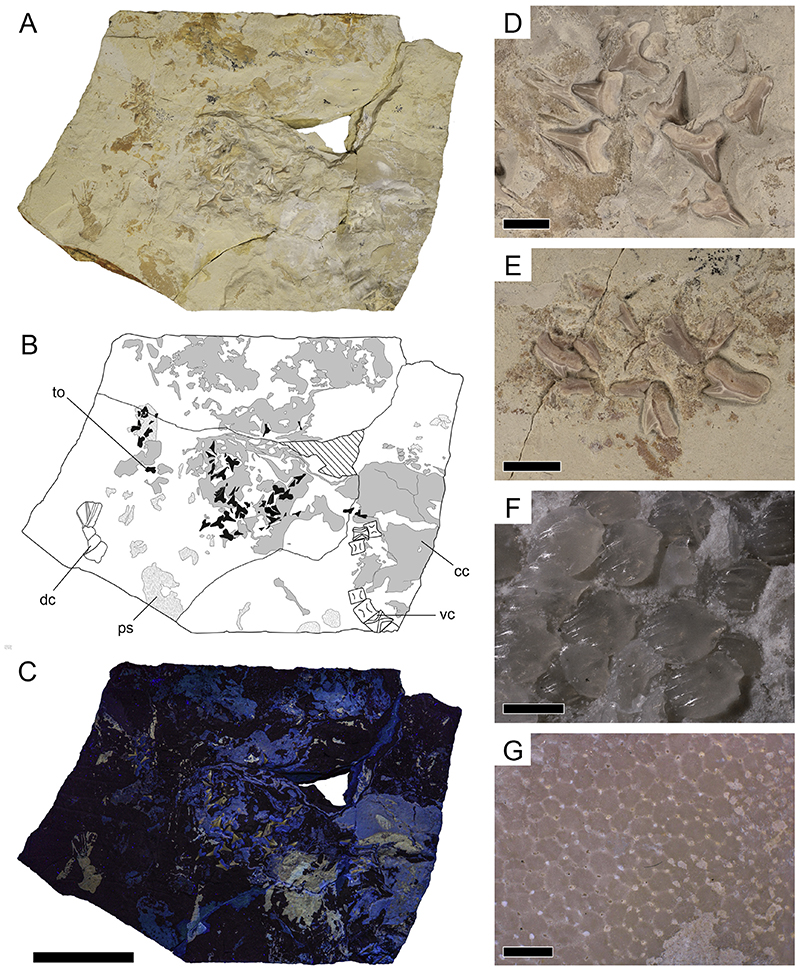
Holotype of *Pseudocorax kindlimanni* sp. nov. (PIMUZ A/I 5037) from the Cenomanian of Haqel, Lebanon (A) under normal light, (B) interpretative drawing, and (C) under ultraviolet light; Close-up images of (D) teeth situated in the center of the slab, (E) teeth situated outside the center, (F) placoid scales, and (G) calcified cartilage composed of tesserae. Abbreviations: cc, calcified cartilage; dc, Decapoda indet.; ps, placoid scales; to, tooth; vc, vertebral centra. Scale bars = 50 mm (A–C), 5 mm (D–E), 0.2 mm (F), 1 mm (G).

The fossil material described herein is deposited and curated in the collection of the Palaeontological Institute and Museum of the University of Zurich, Switzerland (catalogue number: PIMUZ A/I 5037) and currently on display in the ‘Haimuseum und Sammlung R. Kindlimann’ in Aathal-Seegräben, Switzerland. The material consists of an 18 cm × 15 cm limestone slab that preserves an associated set of 70 complete and fragmentary teeth, six slightly disarticulated vertebral centra, placoid scales, and scattered pieces of calcified cartilage. In addition, an indeterminate decapod abdomen is also preserved on the slab (Fig. 2).

Photographs of the specimen were taken with a Nikon D5300 DSLR camera with a mounted AF-S DX Micro NIKKOR 40 mm f/2.8G lens. Detailed images and measurements of the teeth were taken with a Keyence 3D Digital VHX-6000 microscope (Keyence International, Belgium) and a 6-megapixel ZEISS Axiocam 506 Color microscope camera on a ZEISS SteREO Discovery.V20 stereomicroscope (Carl Zeiss Microscopy GmbH, Germany). In addition, photographs were taken under ultraviolet (UV) light following the technique described in Tischlinger and Arratia (2013).

One tooth was extracted from the matrix for non-invasive histological examination using a SkyScan1173 micro-CT device (Bruker/Skyscan, Kontich, Belgium). The scan was performed at 6.08 μm resolution with the following device settings: 100 kV source voltage, 80 μA source current, 750 ms exposure, 0.2° rotation step, and using a 1.0 mm aluminum filter provided with the instrument. To examine and visualize the tooth histology, the resulting stack file was loaded into the software system Amira (version 5.4.5, FEI Visualization Sciences Group, Oregon, USA). Image stacking, colour balance and contrast was adjusted using Adobe Photoshop CS6 (version 13.0, Adobe Systems, San José, USA). All devices utilized for this study are stationed at the Department of Palaeontology (University of Vienna, Austria).

Tooth terminology and systematic classification mainly follows Cappetta (2012). Lamniform sharks exhibit a unique monognathic heterodonty that is often referred to as “lamnoid tooth pattern” (Compagno, 1984; Shimada, 2002). Shimada (2002) established a tooth terminology for lamniform sharks based on the position of the teeth relative to the dental bulla in the lower and upper jaw. The first two mesial files incorporate large anterior teeth and are followed by a file of small intermediate teeth (also called “eye teeth”; Whitley, 1950). These files are situated within the inflated hollow dental bulla. The remaining teeth are situated distally outside the bulla and are referred to as lateral teeth. This tooth terminology was established in order to avoid subjectivity when referring to tooth positions. However, it is only applicable for most macrophagous lamniform sharks, but not for other sharks or lamniforms without a dental bulla, like the extant microphagous basking shark, *Cetorhinus maximus* (Gunnerus, 1765), and megamouth shark, *Megachasma pelagios* Taylor, Compagno & Struhsaker, 1983, or the fossil macrophagous sharks of the family Anacoracidae (Shimada, 2002; Shimada and Cicimurri, 2005). Due to the lack of a typical lamnoid tooth pattern in *P*. *kindlimanni* sp. nov., the tooth terminology was chosen in such a way that it refers to the relative tooth positions within the jaw (“anterior”, “antero-lateral”, “lateral”, and “posterior”), as usually applied to sharks of different orders, but also Lamniformes (e.g., Leriche, 1905; Applegate, 1965; Siversson et al., 2019; Cicimurri et al., 2020). Anatomical terminology of the vertebral centra follows Hasse (1879) and Ridewood (1921).

## 4 Systematic palaeontology

Class Chondrichthyes Huxley, 1880

Subclass Elasmobranchii Bonaparte, 1838, sensu Maisey, 2012

Superorder Galeomorphii Compagno, 1973

Order Lamniformes Berg, 1958

Family Pseudocoracidae Cappetta, 2012

### Type genus

*Pseudocorax* Priem, 1897

*Included genera: Galeocorax* Cappetta, 2012 and *Pseudocorax* Priem, 1897

### Temporal and Spatial Distribution of the family

Late Cretaceous, Cenomanian to late Maastrichtian of Europe, North America, North Africa and the Near East (Cappetta, 2012; this study).

### Diagnosis (emended)

The extinct family Pseudocoracidae includes small lamniform sharks characterized by small, labio-lingually compressed teeth with well-differentiated distal heel adjacent to main cusp; crown labially overhanging the root; tooth root bears a well-marked furrow in which a central foramen opens; well mineralized vertebral centra lacking radial lamellae present.

### Genus

*Pseudocorax* Priem, 1897

Type species: *Corax affinis* Münster in Agassiz, 1843; Late Cretaceous, Maastrichtian of Maastricht, the Netherlands.

### Included species

*Pseudocorax affinis* (Münster in Agassiz, 1843); *Pseudocorax duchaussoisi* Guinot, Underwood, Cappetta & Ward, 2013; *Pseudocorax granti* Cappetta & Case, 1975; *Pseudocorax heteromorphus* (Reuss, 1845); *Pseudocorax laevis* (Leriche, 1906); *Pseudocorax kindlimanni* sp. nov.

### Temporal and Spatial Distribution of the genus

Late Cretaceous, Cenomanian to late Maastrichtian of Europe (Belgium, Czech Republic, Denmark, England, France, Germany, Lithuania, Netherlands, Sweden), North America (Alabama, Delaware, Georgia, Kansas, Mississippi, New Jersey, North Carolina, Texas), North Africa (Morocco), and the Near East (Israel, Lebanon, Syria, Turkey) (Reuss, 1845; Cappetta and Case, 1975; Lauginger and Hartstein, 1983; Case and Schwimmer, 1988; Lewy and Cappetta, 1989; Case, 1991; Case and Cappetta, 2004; Vullo, 2005; Shimada, 2009; Cappetta, 2012; Guinot et al., 2013; Krupp, 2013; Sørensen et al., 2013; van Baal et al., 2013; Adolfssen and Ward, 2014; Cappetta et al., 2014b; Bice and Shimada, 2016; Case et al., 2017; Yilmaz et al., 2018; this study).

### Diagnosis (emended)

Pseudocoracid shark displaying the following unique combination of morphological characters: rather small teeth (measuring up to 15 mm in total tooth height); marked heterodonty developed; anterior teeth with high, upright, and triangular crowns weakly inclined distad; cusps of lateral teeth more strongly distally inclined; crown flanked by two distinct, horizontal or oblique heels; the mesial edge may appear continuous, with no clear separation of a heel; smooth cutting edges in plesiomorphic species but finely serrated in more derived species; convex lingual face of the crown; labial face of cusp is flat; massive root with poorly separated lobes; lateral edges of root lobes straight or slightly concave; no hollow pulp cavity in cross section; well mineralized vertebral centra lacking radial lamellae; placoid scales oval, with flat to rounded crowns; bearing 3–5 longitudinal ridges along the anterior half of the denticle; posterior part smooth.

**Pseudocorax kindlimanni sp. nov**

(urn:lsid:zoobank.org:act:BE7AAD73-E20E-4F1C-ABC0-C4B173442DD4)

(Figs. 2–7)

### Derivation of name

Named in honour of René Kindlimann from Aathal, Switzerland, in recognition of his contribution to the field of palaeontology and for making this specimen available to the scientific community and the public.

### Holotype

PIMUZ A/I 5037, specimen comprising an associated set of 70 teeth, six slightly disarticulated vertebral centra, placoid scales, and scattered pieces of calcified cartilage.

### Type locality and horizon

Sannine Limestone Formation, Haqel, Lebanon (Cenomanian, Upper Cretaceous).

### Diagnosis

The new species of *Pseudocorax* is characterized by the combination of the following dental characters: well-developed heterodonty with distinct anterior, two types of lateral, and discrete posterior teeth; crown with smooth cutting edge; main cusp flanked by two well detached lateral heels; mesial heel well developed and rounded; crown overhanging root with narrow, labio-basal bulge; broad lingual neck separating crown and root present; pulp cavity absent; root bulky and holaulacorhize with lingual protuberance bearing a well-marked nutrient groove; well-developed and symmetrical root lobes on anterior teeth, shorter and laterally expanded root lobes on lateral teeth; root lobes basally flattened.

### Description

**Fig. 3:**
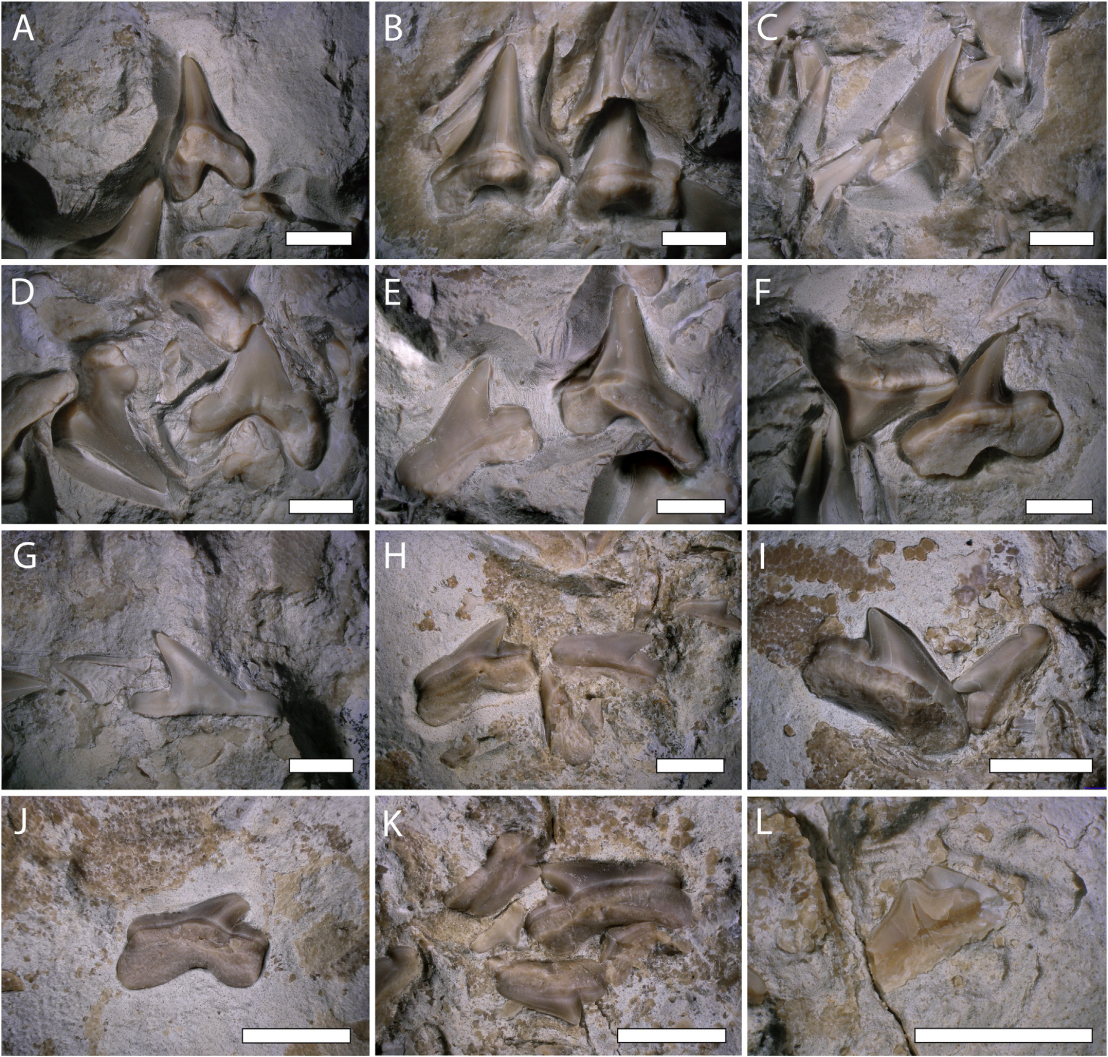
Close-up view images of selected, well-preserved teeth of *Pseudocorax kindlimanni* sp. nov. (PIMUZ A/I 5037). (A) anterior tooth in lingual view; (B) two anterior teeth in lingual view; (C) anterior tooth in labial view; (D) anterior teeth in labial view; (E) antero-lateral tooth in lingual view and lateral tooth (morphotype I) in labial view; (F) lateral teeth (morphotype I) in lingual view; (G) crown of a lateral tooth (morphotype I) in labial view; (H) lateral tooth (morphotype II) in lingual view and lateral tooth (morphotype II) in labial view; (I) lateral teeth (morphotype II) in lingual and labial view; (J) lateral tooth (morphotype II) in lingual view; (K) lateral teeth (morphotype II) in lingual and labial view and one posterior tooth in lingual view; (L) posterior tooth in labial view. Scale bars = 3 mm.

**Fig. 4:**
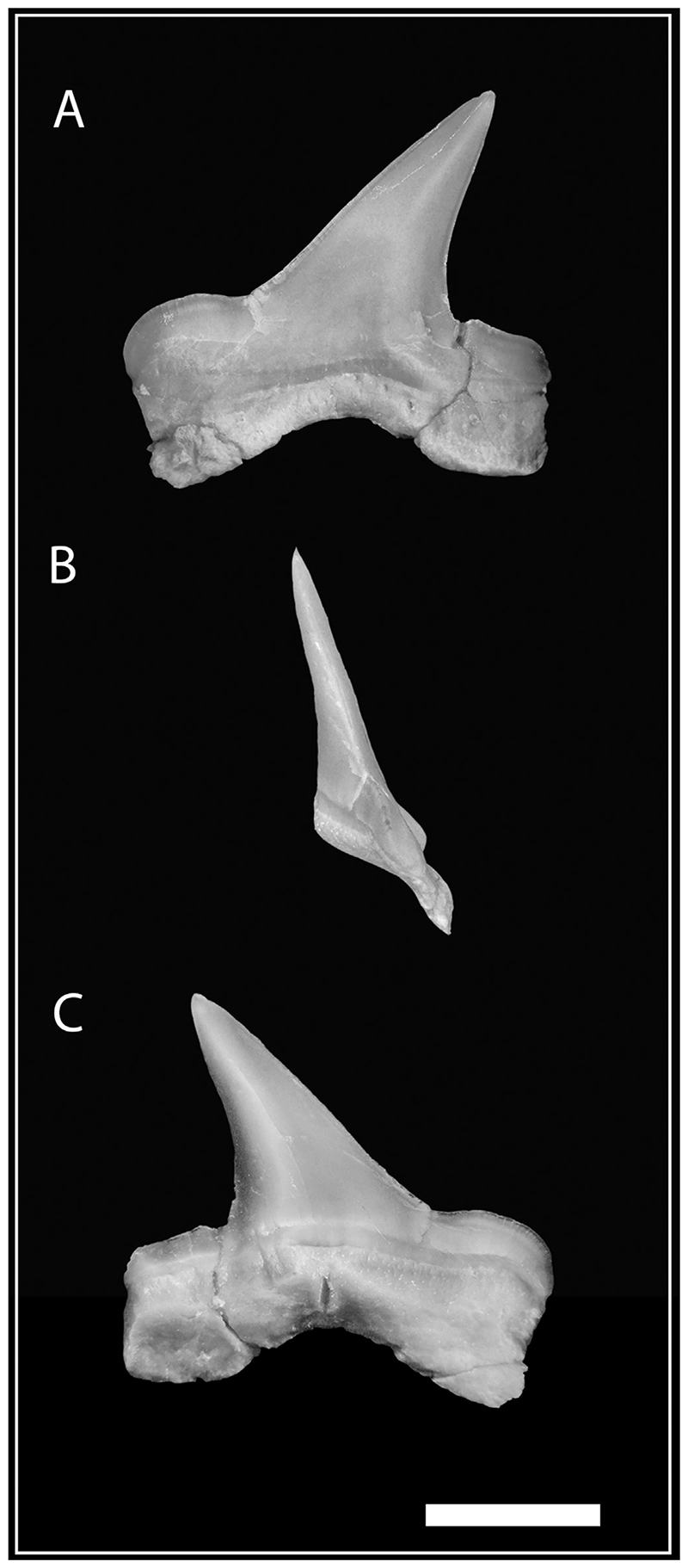
Lateral tooth of *Pseudocorax kindlimanni* sp. nov. (PIMUZ A/I 5037) removed from the matrix. (A) labial view; (B) lateral view; (C) lingual view. Scale bar = 3 mm.

Of the 70 associated teeth preserved in the holotype specimen of *Pseudocorax kindlimanni* sp. nov., 20 teeth are in good to excellent condition, while the remaining teeth are more fragmentary, less mineralized, and often lack the root. The degree of mineralization indicates that the majority of preserved teeth represent nonfunctional replacement teeth. In contrast, the 20 well-preserved teeth have fully mineralized crowns and roots and are considered to be functional teeth (Figs. 3 and 4).

*Pseudocorax kindlimanni* sp. nov. has rather small teeth and exhibits a distinct heterodonty that does not occur in any other species of this genus. Anterior teeth are up to 7.5 mm high and 5.9 mm wide (total height and width respectively), triangular and almost symmetrical with only slightly inclined cusps (75–85° angle) (Fig. 3A-D). The cusp is erect, triangular, slightly bent distally, and flanked by two distinct lateral heels that are oriented perpendicular to the cusp. The mesial heel is separated from the cusp by a shallow notch, whereas the distal heel is more rounded, convex in labial and lingual view, and separated from the main cusp by a deep notch. The cutting edge of the crown is continuous between the main cusp and the heels, smooth and sharp, without any serrations. The apex of the cusp is slightly bent lingually. The labial face of the crown is flat with a mesial depression at the base of the cusp, whereas the lingual surface of the crown is strongly convex and devoid of any ornamentation. Lingually, a prominent neck separates the crown and the root. The labial face of the crown overhangs the root. The holaulacorhize root is strongly bilobate, with two well-developed, basally extended lobes that are more or less symmetrical. The root has an overall bulky appearance but flattens towards the distal end of the root lobes. The lingual face of the root exhibits a well-marked mesial protuberance with a distinct nutrient groove. No intermediate tooth (“eye tooth”; Whitley, 1950) could be identified. Lateral teeth display two distinct morphotypes that probably reflect their relative position within the jaw (antero-lateral and latero-posterior), and are herein subsequently referred to as morphotype I and II (Figs. 3 and 4). Morphotype I teeth are almost as high as wide (up to 6.6 and 7.4 mm total height and width respectively). The teeth are asymmetrical, with distally directed cusps (55–75° angle) that become progressively more inclined towards the jaw commissure (Figs. 3E-G and 4). The apex of the crown is slightly bent lingually. The lingual face is convex and the labial face flattened but with a basal mesial depression. The lateral heels flanking the main cusp are oriented perpendicular to, and are well separated from, the cusp by a quite deep notch. The mesial heel is more developed and strongly convex in labial view. The outline of the distal heel is either rectilinear or slightly convex, but never to the same extent as in the mesial heel. The crown overhangs the root labially, and lingually a neck separates the crown from the root. The root lobes are more divergent and asymmetrical, with the mesial root branch being more expanded, whereas the distal branch is shorter and slightly more massive. The root lobes coalesce in their upper part to form a well-developed, concave arch. On the labial side of the root, several foramina are arranged below the crown along the arch-like contour of the upper root portion.

Teeth corresponding to morphotype II are wider than high (up to 3.4 and 5.7 mm total height and width respectively), asymmetrical, with an acutely recurved main cusp (40–50° angle) (Figs. 3H-K). The teeth are labio-lingually compressed with slightly convex lingual and labial faces that are devoid of any ornamentation. The apex of the crown is straight, showing no lingual bend like in anterior or morphotype I teeth. Both lateral heels still are developed, but the mesial heel can be less distinctive or even merge completely with the main cusp to form a continuous cutting edge. The distal heel is less developed than the mesial heel, but always detached from the main cusp by a clear notch. Its apical cutting edge is concave, resulting in a more or less sinuous outline of the distal heel. Labio-basally, the crown juts out over the root. The root bears a lingual protuberance that is less prominent than on anterior teeth and teeth of morphotype I, and restricted to the upper part of the root, close to the neck. The root lobes are flattened. A nutrient groove is present. The root lobes are very short, rather symmetrical with flattened lower extremities, and they meet apically to form a gently arched concavity.

Three teeth can be referred to the posterior-most position based on their small size and morphology (Fig. 3K-L). They are wider than high (up to 1.5- and 2.2-mm total height and width respectively), asymmetrical, with a slightly bent main cusp (80–90° angle). The mesial and distal cutting edges are not very distinctive in these teeth, and are either weakly detached from the main cusp or coalesced with it completely, forming an incipient triangular shape. Both the labial and lingual crown faces are slightly convex. The labial face overhangs the root. The basal root face is flat, with two almost horizontal and not well-separated branches, of which the mesial one is slightly better developed than the distal one.

**Fig. 5:**
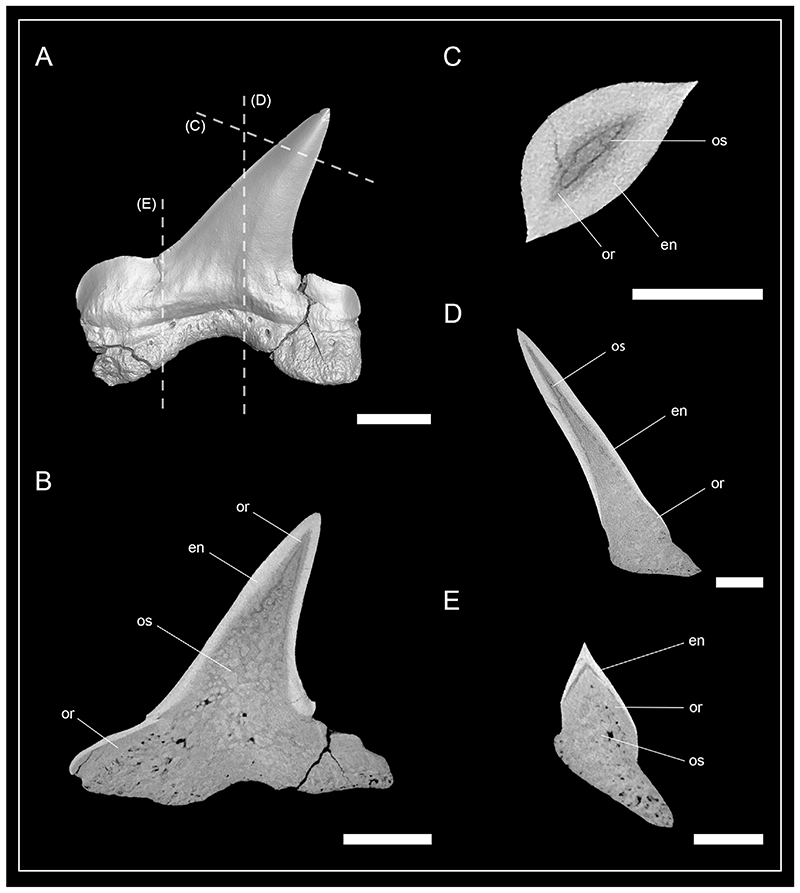
Micro-CT images and tooth histology of *Pseudocorax kindlimanni* sp. nov. Isosurface (A) and virtual tooth sections through the (B) frontal, (C) axial, and (D, E) sagittal plane of a lateral tooth. Sagittal sections are through the (D) main cusp and the (E) mesial heel. Abbreviations: en, enameloid; or, orthodentine; os, osteodentine. Scale bars = 2 mm (A–B), 1 mm (C–E).

Teeth of *Pseudocorax kindlimanni* sp. nov. lack a hollow pulp cavity and instead have a solid dentine core (Fig. 5). This dentine core is traversed by numerous dentinal osteons that are distributed throughout the root and the majority of the crown. This results in a bone-like appearance of the dentine, which is characteristic for osteodentine. A hypermineralized layer of enameloid covers the tooth crown and is distinguishable from the dentine by its higher density, giving it a brighter appearance in the micro-CT scan (seen in white). Orthodentine also occurs in the tooth crown, which is composed of numerous tiny parallel tubules that are not detectable in the micro-CT scan, giving it a compact appearance. The mesial and distal heels contain a prominent layer of orthodentine that lies between the osteodentine core and the superficial enameloid. Within the main cusp a prominent layer of orthodentine is restricted to the basal portion of the crown (Fig. 5B and E), but the layer becomes narrower apically, where it is only visible as a very thin layer (Fig. 5B-D).

Specimen PIMUZ A/I 5037 is the first pseudocoracid shark fossil preserving teeth, some anterior-most vertebral elements, scattered placoid scales, and pieces of calcified cartilage that most likely represent cranial and/or branchial elements (Fig. 2A-C). The anatomical identity of these remains, however, cannot be established due to their fragmentary nature.

**Fig. 6:**
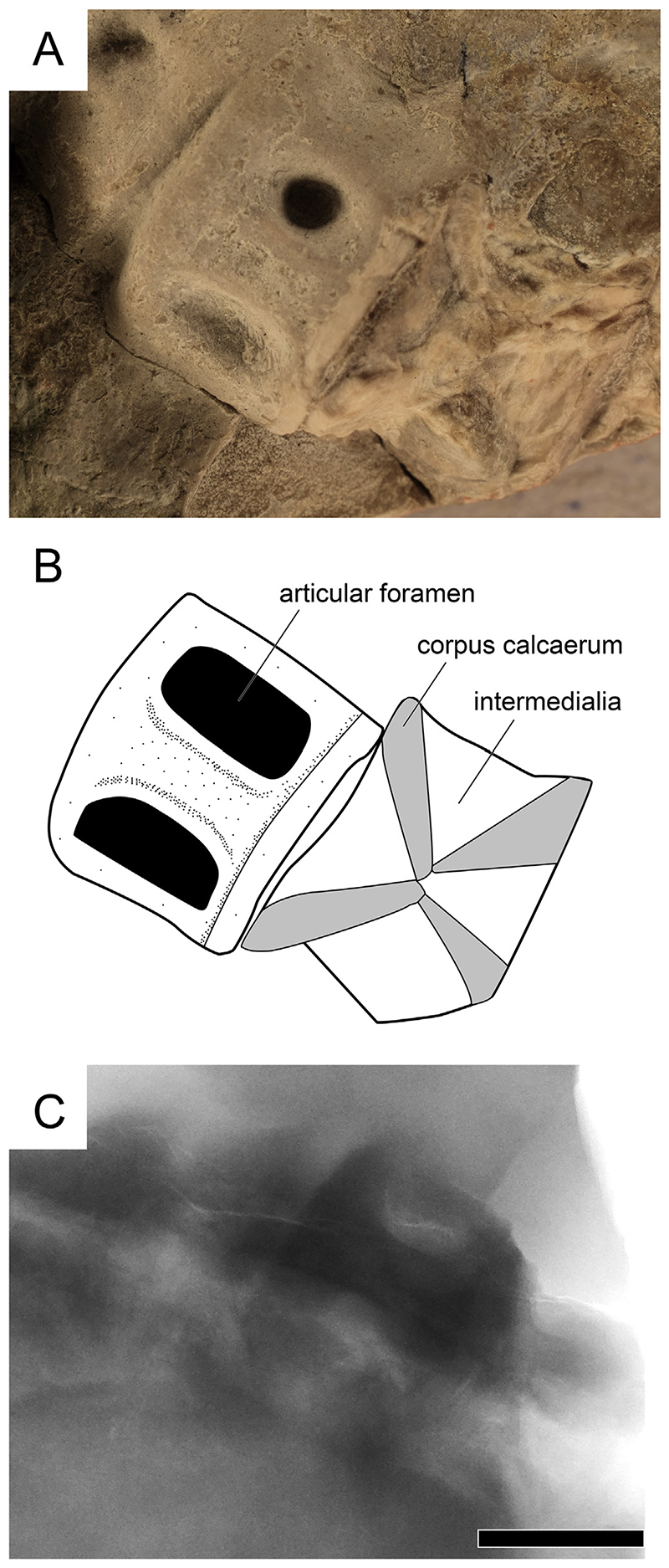
Vertebral centra of *Pseudocorax kindlimanni* sp. nov. (A) Close up photograph, (B) illustration, and (C) X-ray image of the fifth and sixth vertebral centra showing a pair of articular foramina without radiating calcified lamellae between the two articular faces of the centra. Scale bar = 5 mm.

The vertebral centra are well-mineralized, with deep amphicoelous (biconcave) anterior and posterior articulation surfaces. Of the six preserved vertebral centra, most are fragmentary or distorted, however, V5 is well-preserved (Figs. 2 and 6). It is taller than long (11.4 mm × 8.1 mm), shows two well-developed foramina on the exposed surface, but lacks radiating calcified lamellae typical for lamniform sharks in the area between the two articular faces (referred to as intermedialia) and instead resembles the vertebral centrum morphology known from many carcharhiniform sharks (Fig. 6).

**Fig. 7:**
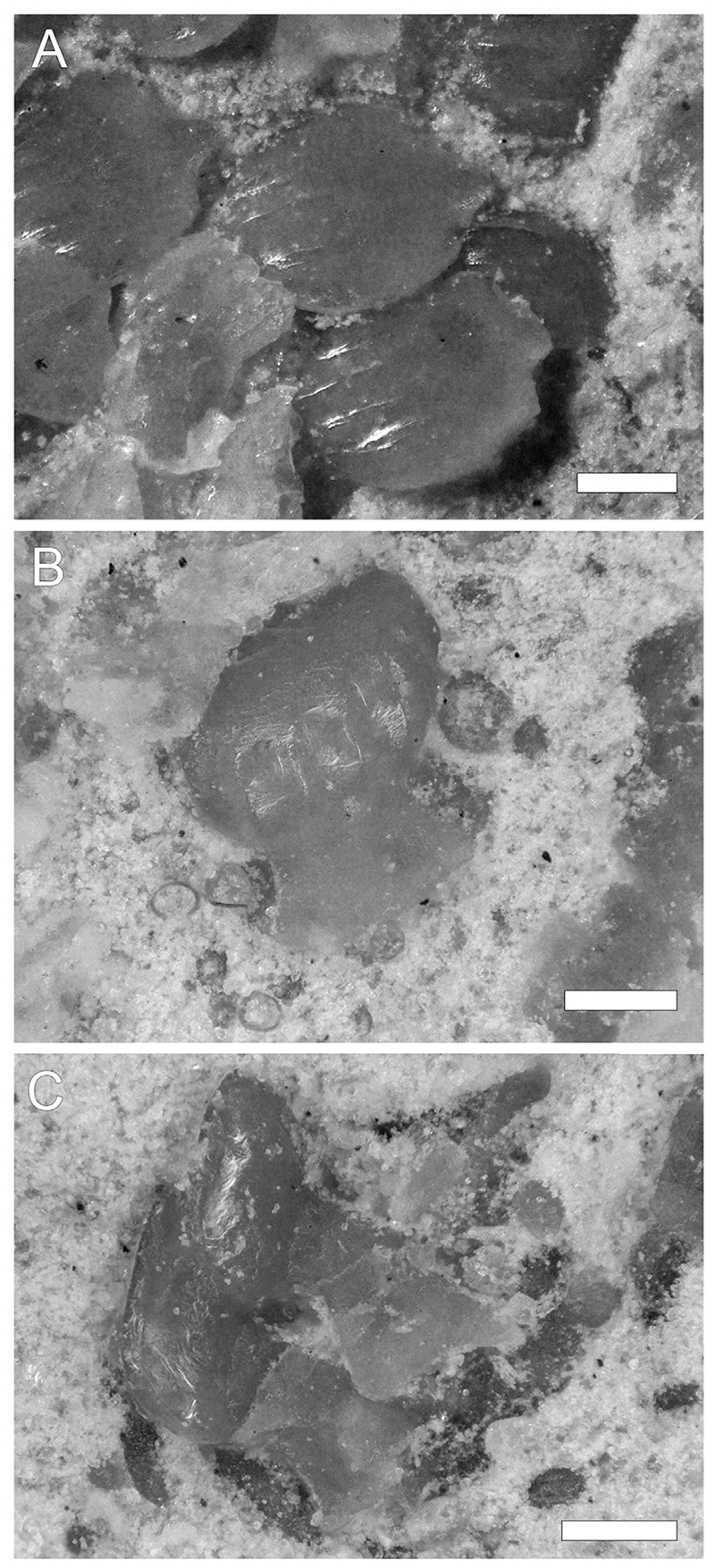
Placoid Scales of *Pseudocorax kindlimanni* sp. nov. (PIMUZ A/I 5037). (A) apical view; (B) frontal view; (C) lateral view. Scale bars = 100 μm.

Placoid scales are scattered or loosely organized in patches across the slab. They are up to ca. 280 μm high, with an anteriorly inclined oval crown that is up to 275 μm long and 230 μm wide. The crown surface is flat to gently rounded and bears three to six longitudinal ridges that are restricted to the anterior half of the crown (Fig. 7). The interkeel distances between these ridges range from 30 to 50 μm.

### Remarks

Six species of *Pseudocorax* are currently considered valid: *P*. *affinis* (Campanian–Maastrichtian; Case et al., 2017 and references therein), *P*. *duchaussoisi* (Turonian–Coniacian; Guinot et al., 2013), *P*. *granti* (Coniacian–Maastrichtian; Cappetta 2012), *P*. *heteromorphus* (Turonian; Cappetta, 2012), *P*. *laevis* (Turonian–Campanian; Cappetta, 2012), and *P*. *kindlimanni* sp. nov. (Cenomanian). Hamm and Shimada (2007) stated that diagnostic characters used to separate *P*. *granti* from *P*. *laevis* are weakly founded and proposed that these taxa could be regarded as conspecific, with *P*. *laevis* having priority. This hypothesis requires further testing.

Müller and Diedrich (1991) described the pseudocoracid shark *P*. *primulus* from the Cenomanian of Ascheloh, Teutoburger Wald, Germany. However, this species lacks the typical lingual furrow at the root, which is a defining character for the family Pseudocoracidae that separates it from the morphologically similar teeth of members of the family Anacoracidae. The lack of this furrow has led to the reassignment of *P*. *primulus* to either of the anacoracid genera *Squalicorax* (Siverson et al., 2007; Cappetta et al., 2014a; Siversson et al., 2019), or *Palaeoanacorax* (Underwood and Cumbaa, 2010).

Another species, *P*. *heteromorphus*, was described from the Turonian Pläner limestones of Koštice (Kosstitz), Czech Republic by Reuss (1845). The author originally described these teeth as *Oxyrhina heteromorpha* (“*Oxyrrhina heteromorpha*”) but reassigned them to *Scoliodon priscus* in the addendum of the same work. Further material ascribed to this species was collected in Novosedlice (Weisskirchlitz), Czech Republic (Reuss, 1845) and Plauen, Dresden, Germany (Fischer, 1856). Giebel (1848) transferred this species to *Carcharias priscus* (also see: Fritsch, 1878; Žítt and Vodrázka, 2013; Žítt et al., 2015, 2019), while Geinitz (1875) proposed that teeth assigned to *O*. *heteromorpha* and *O*. *acuminata* represent different tooth positions of the same species and regarded them to be synonymous with *O*. *angustidens* (now *Paranomotodon angustidens*), an assumption that was followed by subsequent studies (e.g., Eastman, 1895; Woodward, 1911; Herman, 1975; Müller and Diedrich, 1991). Cappetta (2012) reintroduced the species and assigned it to the family Pseudocoracidae as *P*. *heteromorphus*, but without giving any justification for the resurrection and assignment of this species. Unfortunately, the type material of *O*. *heteromorpha* appears to be lost. The Natural History Museum of Vienna owns parts of the Reuss collection, which does also include teeth of *Oxyrhina heteromorpha* (catalogued under NHMW 1864 XL 9, NHMW 1864 XL 10, NHMW 1864 XL 11) and *Scoliodon priscus* (NHMW 1864 XL 26), although not the type material. Specimen NHMW1864 XL 9 and NHMW1864 XL 26 are from the type locality (Koštice/Kosstitz), but only the crowns are preserved which does not allow a clear identification of the species. Although there is more material of *P*. *heteromorphus* from the Czech Republic (PLJ, pers. obser.), it is in a private repository which cannot be used to confirm its status. It also should be noted that *P*. *heteromorphus* shows a high degree of similarity to the French species, *P*. *duchaussoisi*. Unfortunately, Guinot et al. (2013) did not compare both species, so it remains unclear as to whether these two contemporary species are conspecific, in which case *P*. *heteromorphus* (Reuss, 1845) would have priority over *P*. *duchaussoisi* Guinot, Underwood, Cappetta & Ward, 2013. Reviewing the whole group of these enigmatic sharks is beyond the scope of this manuscript, but is certainly warranted in the future for a better understanding of the taxonomic composition of the genus. For the purpose of this report, we tentatively regard both *P*. *heteromorphus* and *P*. *duchaussoisi* to represent two distinct and valid species.

The tooth morphology of *Pseudocorax kindlimanni* sp. nov. is easily distinguishable from other *Pseudocorax* species by the very distinctive, well-developed and detached lateral heels, particularly the very prominent, rounded mesial heel that occurs on anterior and most lateral teeth, except for some teeth of the posteriormost tooth files. Besides these differences in the crown morphology, *P*. *kindlimanni* can be distinguished from *P*. *laevis* by the gently rounded basal concavity between the root lobes, which is V-shaped in latter species. *Pseudocorax granti* exhibits a generally more gracile crown and root morphology compared to *P*. *laevis* and *P*. *kindlimanni* sp. nov. Its lingual face is moderately convex (strongly convex in *P*. *kindlimanni* sp. nov.) and the root branches are long and thin (shorter and laterally expanded in P. kindlimanni sp. nov.). The lack of serrations on teeth of *P*. *kindlimanni* sp. nov. makes this species easily distinguishable from the Maastrichtian species *P*. *affinis*. The tooth size and morphology of the new species are most similar to the Turonian*P*. *duchaussoisi* from France (Guinot et al., 2013). Teeth of *P*. *kindlimanni* sp. nov. differ from those of *P*. *duchaussoisi* by their prominent mesial heels that form a convex cutting edge, whereas those of *P*. *duchaussoisi* are less developed and rectilinear. Moreover, the root of *P*. *kindlimanni* sp. nov. is less bulky and basally more flattened than on *P*. *duchaussoisi*. The root lobes are either equally developed or asymmetric, with the mesial root branch being laterally expanded in *P*. *kindlimanni* sp. nov. The root shows a lesser degree of asymmetry, with mesial branches being slender and less distinct in *P*. *duchaussoisi*. Overall, the root extremities of lateral teeth are more pointed in *P*. *duchaussoisi* than in *P*. *kindlimanni* sp. nov. The basal concavity of the root between the lobes is gently arched, shallow, and rounded on teeth of *P*. *kindlimanni* sp. nov., but it is more V-shaped in *P*. *duchaussoisi*. It should be noted here that *P*. *kindlimanni* sp. nov. appears to exhibit a higher degree of heterodonty compared to *P*. *duchaussoisi*, which has teeth showing similarities with anterior and lateral teeth of morphotype I, but morphotype II is not known in the latter species (this could be the result of incomplete preservation). The posterior teeth of *P*. *kindlimanni* sp. nov. also differ significantly from those of *P*. *duchaussoisi* in having less distinct heels, narrower main cusps, and more compact overall morphology. The basal concavity between the root lobes is almost completely reduced, the root branches are horizontal and barely separated in *P*. *kindlimanni* sp. nov., which is in stark contrast to the posterior teeth of *P*. *duchaussoisi* that still have high similarity to the lateral teeth (and to some degree to morphotype I of *P*. *kindlimanni* sp. nov.), but exhibit gently reduced roots.

## 5 Discussion

### 5.1 Heterodonty and dental pattern of Pseudocorax kindlimanni *sp. nov*

Only a single associated tooth set, belonging to *Pseudocorax laevis* and consisting of four teeth, has been previously described for the family Pseudocoracidae (Shimada, 2009). The holotype specimen of *P*. *kindlimanni* sp. nov. represents the most complete fossil of this family to date, as it consists of 70 teeth, six vertebral centra, placoid scales, and pieces of calcified cartilage. Species of *Pseudocorax* were previously assumed to exhibit heterodonty, but the degree of heterodonty has remained unclear. *Pseudocorax kindlimanni* sp. nov. exhibits a high degree of heterodonty, with erect, almost symmetrical anterior teeth, whereas the cusps in lateral teeth are successively more recurved. We recognized two distinct morphotypes of lateral teeth. In morphotype I, the mesial heels are well-detached from the cusp, but on morphotype II the mesial heel merges with the main cusp to form a continuous mesial cutting edge. Morphotype II teeth are smaller and the cusp is more distally bent than in morphotype I teeth, indicating that they were located on the same jaw ramus, with morphotype I representing antero-lateral teeth and morphotype II postero-lateral teeth. This dental pattern would indicate a monognathic heterodonty in *Pseudocorax*. It is, nevertheless, not possible to determine if and to what extent dignathic heterodonty was developed in this species because of the disarticulated nature of the specimen. However, the lack of additional morphotypes of similar size indicates that *P*. *kindlimanni* sp. nov. did not exhibit a marked dignathic heterodonty.

A unique heterodonty is known for lamniform sharks that is referred to as “lamnoid tooth pattern” (Compagno, 1984; Shimada, 2002). This tooth pattern comprises two files of large anterior teeth that are followed by one or more files of small intermediate teeth (“eye teeth”) and several files of lateral teeth. Intermediate teeth are easily recognized by their reduced size compared to the preceding anterior teeth and succeeding lateral teeth. This pattern occurs in almost all extant macrophagous lamniform sharks (all except for the bigeye thresher, *Alopias superciliosus*) and several fossil taxa, but not in the filter feeding basking shark (*Cetorhinus maximus*) and megamouth shark (*Megachasma pelagios*), or the extinct macrophagous anacoracid sharks and *Haimirichia amonensis* (Shimada, 2002; Shimada and Cicimurri, 2005; Vullo et al., 2016). We were not able to identify intermediate teeth in *P*. *kindlimanni* sp. nov., indicating that pseudocoracid sharks did not exhibit a lamnoid tooth pattern. However, it must be noted that the teeth of PIMUZ A/ I 5037 were disarticulated and the intermediate tooth might simply have got lost postmortem.

### 5.2 Implications of the tooth histology in Pseudocorax kindlimanni *sp. nov*

Dental histology has long been proposed to be one of the most reliable characters to distinguish between higher systematic categories of chondrichthyans (Cappetta, 2012). Differences in the enameloid microstructure (Enault et al., 2015; Cuny et al., 2017) and dentine composition of the crown (Glickman, 1964; Jambura et al., 2020) previously have been used to distinguish between shark groups of different taxonomic levels. Originally, two tooth histology patterns (“histotypes”) were distinguished: (1) orthodont histotype in teeth bearing a hollow pulp cavity surrounded by orthodentine and (2) osteodont histotype in teeth that have no hollow pulp cavity but instead a crown that is filled by osteodentine but lacks orthodentine (Glickman, 1964; Moyer et al., 2015). A third term, pseudoosteodonty was introduced to distinguish between osteodont teeth that are solely composed of osteodentine (osteodont teeth) and teeth in which the hollow pulp cavity is secondarily infilled with osteodentine from the root, but a layer of orthodentine still encapsulates the osteodentine core (Herman et al., 1991; Jambura et al., 2018). The order Lamniformes are the only known group (except for the Palaeozoic chondrichthyan *Aztecodus harmsenae* (Hampe and Long, 1999)) having teeth that are solely composed of osteodentine (Moyer et al., 2015; Schnetz et al., 2016; Jambura et al., 2019). Morphology of *Pseudocorax* teeth is similar to that of hammerhead sharks (Sphyrnidae), but they differ from them histologically by having teeth that are fully filled with dentine (Priem, 1897). This led Priem (1897) to assume that *Pseudocorax* might be closely related to the lamniform shark family Anacoracidae. Our examination of the tooth histology in *P*. *kindlimanni* sp. nov. confirms the lack of a hollow pulp cavity, but orthodentine is also present. Therefore, *P*. *kindlimanni* sp. nov. exhibits the pseudoosteodont tooth histotype, deviating from the osteodont tooth histotype typically found in lamniform sharks. Nevertheless, this does not necessitate the exclusion of *Pseudocorax* from this group, as pseudoosteodonty occurs in a number of shark taxa including the lamniform basking shark *Cetorhinus maximus* (Jambura et al., 2019). Furthermore, the taxonomic value of tooth histology patterns was recently questioned because, in addition to a phylogenetic signal, tooth morphology (functional aspect of the tooth) could heavily influence the development of different mineralization patterns in sharks (Jambura et al., 2020).

### 5.3 Palaeoecology of Pseudocorax kindlimanni *sp. nov*

Based on the size of the teeth (up to 15 mm high), members of the genus *Pseudocorax* are generally regarded as having been small sharks with an estimated total body length (TL) of ca. 100 cm (Hamm and Shimada, 2007; Cappetta, 2012). In an attempt to quantify the body size distribution of lamniform sharks through geological time, Shimada et al. (2020) generated functions based on the 13 extant macrophagous representatives of this group to predict body, jaw, and dentition lengths based on the crown height (CH). They were able to show strong quantitative relationships between these measurements and achieved estimates that were in accordance with known holomorphic material. According to their estimations, pseudocoracid sharks reached a maximum TL of 106 cm (*Galeocorax*) and 129 cm (*Pseudocorax*) respectively. Using their formula (TL = 11.784 × CH - 0.331) we estimated a TL of 75 cm (CH = 6.4 mm) for *P*. *kindlimanni* sp. nov. It is important to keep in mind that this estimation is based on a single specimen of unknown ontogenetic stage, and the existence of larger individuals cannot be ruled out. Nonetheless, pseudocoracid sharks from the Turonian are known to have similar-sized teeth, and we therefore presume that specimen PIMUZ A/I 5037 represented an adult individual of at least average size.

Although the known material of *P*. *kindlimanni* sp. nov. does not allow us to draw any direct conclusions about body shape or swimming performance, some inferences are possible based on the morphology of the preserved dermal denticles (placoid scales). Placoid scales are considered to fulfill several important functions, including protection from ectoparasites, reduction of mechanical abrasion, accommodation of bioluminescent organs, and drag reduction (Raschi and Tabit, 1992). They also can shelter sensorial organs, e.g., highly specialized dermal denticles embedding ampullary organs are present in the extinct lamniform shark *Haimirichia amonensis* (Vullo and Guinot, 2015; Vullo et al., 2016). Consequently, a plethora of different scale morphologies exists among sharks, and even individual sharks show a high degree of variation along different parts of the body. Generalizations about the relationship between form and function of these denticles are therefore difficult to make. Nonetheless, theoretical and experimental studies have shown that placoid scales with a keeled surface reduce drag and thus can facilitate a more efficient swimming performance (Walsh 1983; Bechert et al., 1985; Oeffner and Lauder, 2012). The morphology, size and interkeel distance (<100 μm) of the placoid scales preserved in *P*. *kindlimanni* sp. nov. are comparable to those of fast-swimming sharks. However, contrary to active pelagic sharks like makos (*Isurus* spp.) or hammerheads (*Sphyrna* spp.), the ridges of the placoid scales in *P*. *kindlimanni* sp. nov. do not extend along the entire length of the denticle but are instead confined to the anterior half of the cap. This indicates placoid scales of *P*. *kindlimanni* sp. nov. provided some drag reduction, but to a lesser extent than scales on fast swimming species. We hypothesize that *P*. *kindlimanni* sp. nov. was a rather sluggish shark that was capable of much higher swimming speeds during short bursts. It should be noted that the examined placoid scales of *P*. *kindlimanni* sp. nov. all came from the anterior, cranial region, and the scale morphology in the trunk and caudal region remains unknown. Nonetheless, our inferences about the swimming performance of *P*. *kindlimanni* sp. nov. seem justified based on the fact that the aforementioned taxa exhibit similar scale morphologies in the head, trunk and caudal region (Dean and Bhushan, 2010). *Cretoxyrhina mantelli* (Agassiz, 1835), a large extinct lamniform shark that was widespread during the Late Cretaceous, is often regarded to ecomorphologically represent the modern white shark, *Carcharodon carcharias* (Linnaeus, 1758), including being a fast swimmer (Shimada, 1997; Amalfitano et al., 2019). *Cretoxyrhina mantelli* is known to have had both placoid scales with ridges that are confined to the anterior part, and scales where the ridges extend to the posterior margin (Amalfitano et al., 2019). This further supports the notion that *P*. *kindlimanni* sp. nov. was an active, predatory shark capable of fast swimming maneuvers.

The Cretaceous was an important time in the evolution of lamniform sharks, as the group underwent major radiations during the Aptian and Cenomanian (Underwood, 2006; Guinot et al., 2013; Guinot and Cavin, 2016). This rapid diversification resulted in the appearance of a number of fossil and extant clades and gave rise to a variety of different specializations and ecomorphotypes, like the small nectobenthic predator *Haimirichia amonensis*, that resembled modern reef sharks (Vullo et al., 2016). It was also during this time when *Pseudocorax* first appeared, possessing a set of ecological and biological traits not known from other extinct or extant lamniform sharks. The dentition of *P*. *kindlimanni* sp. nov. includes a clutching morphotype in the anterior portion, whereas the lateral teeth with their strongly bent cusps represent a cutting morphotype. This phenomenon was also reported for *H. amonensis* (Vullo et al., 2016), but it is developed to a lesser degree than in *Pseudocorax*. The strong monognathic heterodonty, with clutching and cutting type dentition, indicates that *Pseudocorax* was a generalist feeder. Extant sharks with a comparable tooth morphology (e.g., hammerhead sharks, *Sphyrna* spp. and the school shark *Galeorhinus galeus* (Linnaeus, 1758)) are known to feed on a range of different prey items, including cephalopods, crustaceans, and bony fishes (Ebert and Dando, 2020), and we therefore presume a similar generalist lifestyle for *P*. *kindlimanni* sp. nov. Small shark bite marks on a tylosaurine mosasaur skeleton from the Ozan Formation (late Campanian) of Texas were referred to *P*. *laevis*, indicating that scavenging might have also occurred in this group (Hamm and Shimada, 2007).

## 6 Concluding remarks

In this study we described a new species of *Pseudocorax* from the Cenomanian of Haqel, Lebanon. *Pseudocorax kindlimanni* sp. nov. is the oldest known pseudocoracid shark, extending the fossil record of this genus and family further back in time. The described specimen is also the most complete fossil of this group known to date, and it provides important information about the dental pattern and other anatomical characters in this species. The associated tooth set indicates that this shark did not exhibit the lamnoid tooth pattern observed in other macrophagous lamniform sharks. In addition, the tooth histology and the morphology of the vertebral centra deviate from what is considered “characteristic” for lamniform sharks. Nonetheless, other lamniform sharks are known to exhibit these features (e.g., the basking shark, *Cetorhinus maximus*), and the allocation of *Pseudocorax* to another order is not warranted. *Pseudocorax kindlimanni* sp. nov. is interpreted to represent a small mesopredator occupying the lower trophic levels of the early Late Cretaceous marine food web. It was capable of fast swimming and actively hunted, but likely also opportunistically scavenged. Our findings provide novel insights into the early evolution of pseudocoracid sharks and shed additional light on the diversity and radiation of lamniform sharks during the early Late Cretaceous.
